# A182 PREDICTING HIGH DIRECT HEALTHCARE COSTS IN PEDIATRIC PATIENTS WITH INFLAMMATORY BOWEL DISEASE IN THE FIRST YEAR FOLLOWING DIAGNOSIS

**DOI:** 10.1093/jcag/gwac036.182

**Published:** 2023-03-07

**Authors:** E Kuenzig, R Duchen, T D Walters, D R Mack, A M Griffiths, C N Bernstein, G G Kaplan, A R Otley, W Yu, X Wang, J Guan, S Fung, E I Benchimol

**Affiliations:** 1 Child Health Evaluative Sciences, SickKids Research Institute; 2 SickKids Inflammatory Bowel Disease Centre, Division of Gastroenterology, Hepatology and Nutrition, The Hospital for Sick Children; 3 ICES, Toronto; 4 Pediatrics, University of Ottawa; 5 CHEO Inflammatory Bowel Disease Centre, Division of Gastroenterology, Hepatology and Nutrition, CHEO; 6 CHEO Research Institute, Ottawa; 7 Paediatrics, University of Toronto, Toronto; 8 Univeristy of Manitoba IBD Clinical and Research Centre; 9 Department of Internal Medicine, Max Rady College of Medicine, , University of Manitoba, Winnipeg; 10 Medicine & Community Health Sciences, University of Calgary, Calgary; 11 Pediatrics, Dalhousie University, Halifax; 12 Institute of Health Policy, Management and Evaluation, University of Toronto, Toronto, Canada

## Abstract

**Background:**

The incidence of inflammatory bowel disease (IBD) continues to rise rapidly among Canadian children. The care of children results in higher direct healthcare costs than adults with IBD. It is imperative that we identify individuals who will become the highest-cost users of the health system in order to intervene early and decrease the individual- and system-level burden of IBD.

**Purpose:**

To develop a predictive-model for high-cost health system users and (2) identify factors associated with high-cost healthcare use.

**Method:**

Incident cases of IBD diagnosed ≤17y residing in Ontario and enrolled in the Canadian Children IBD Network (CIDsCaNN) between Dec 31 2013 and Jan 31 2019 were linked deterministically using health card number to health administrative data. Using a validated algorithm, direct healthcare costs accumulated between the 31^st^ and 365^th^ day after diagnosis were calculated using data from CIDsCaNN (medications) and health administrative data (health system encounters, including surgery). A predictive model was created to determine high-cost (≤25^th^ percentile) and medium-cost (26^th^ to 75^th^) users, compared to low-cost users (>75th) using ordinal logistic regression. Potential predictive variables were determined *a priori* based on clinical significance and magnitude of univariable association, based on sample size-informed degrees of freedom. Variables from CIDsCaNN data included: IBD type, age at diagnosis, sex, first line of therapy (steroids, aminosalicylates, exclusive enteral nutrition; yes or no, not mutually exclusive), disease activity (severe vs. moderate vs. none/mild based on PUCAI [UC] or wPCDAI [Crohn’s]). Predictive variables from the health administrative data included: rural/urban residence, hospitalization at diagnosis, intestinal resection or colectomy within 3 months of diagnosis, emergency department visit ±1 month of diagnosis, and a mental health encounter within the first year following diagnosis. Anti-TNF treatment was excluded from models due to the strong correlation with the outcome (direct costs). Overall model fit was estimated with a c-statistic.

**Result(s):**

Among the 487 (57% Crohn’s) children included in the study, the mean (sd) direct costs accumulated between the 31^st^ and 365^th^ days following IBD diagnosis was $14,451 (14,665). The mean cost among high-cost users was $33,533 (16,530); medium-cost users, $11,038 (5322); low-cost users, $2530 (831). The predictive model identified high-cost users of the health system with acceptable model fit (c-statistic 0.69). The relative contribution of individual variables, as measured by odds ratio (OR), is reported in the Table.

**Image:**

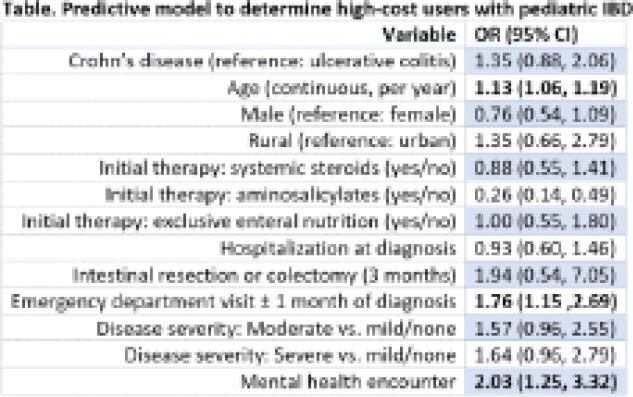

**Conclusion(s):**

The direct healthcare costs of pediatric IBD are substantial. Children with IBD who become high-cost users of the health system were identifiable using characteristics at diagnosis (e.g., need for mental health care, emergency visits, older age). Further research should assess whether interventions in patients at-risk for becoming high-cost users may help to reduce costs.

**Please acknowledge all funding agencies by checking the applicable boxes below:**

Other

**Please indicate your source of funding;:**

Ontario Academic Health Sciences Centres Alternate Funding Plan Innovation Fund

**Disclosure of Interest:**

E. Kuenzig: None Declared, R. Duchen: None Declared, T. Walters Grant / Research support from: Janssen, Abbvie, Psfizer, Ferring, Amgen, Consultant of: Janssen, Abbvie, Psfizer, Ferring, Amgen, D. Mack: None Declared, A. Griffiths Grant / Research support from: Abbvie, Consultant of: Abbvie, Amgen, BristolMyersSquibb, Janssen, Lilly, Takeda, Speakers bureau of: Abbvie, Janssen, Takeda, C. Bernstein Grant / Research support from: Research grants from Abbvie Canada, Amgen Canada, Pfizer Canada, and Sandoz Canada and contract grants from Janssen, Abbvie and Pfizer, Consultant of: Abbvie Canada, Amgen Canada, Bristol Myers Squibb Canada, JAMP Pharmaceuticals, Janssen Canada, Pfizer Canada, Sandoz Canada, Takeda, Speakers bureau of: Abbvie Canada, Janssen Canada, Pfizer Canada and Takeda Canada, G. Kaplan Grant / Research support from: Ferring, Consultant of: AbbVie, Janssen, Pfizer, Amgen, Sandoz, Pendophram, and Takeda, Speakers bureau of: AbbVie, Janssen, Pfizer, Amgen, Sandoz, Pendophram, and Takeda, A. Otley Grant / Research support from: Research support: AbbVie Global. Research site: AbbVie, Pfizer, Eli-Lily, Janssen, Consultant of: AbbVie Canada, W. Yu: None Declared, X. Wang: None Declared, J. Guan: None Declared, S. Fung: None Declared, E. Benchimol Consultant of: McKesson Canada, Dairy Farmers of Ontario (unrelated to medications used to treat inflammatory bowel disease)

